# Lucilia Macellaria—“Texas Screw Worm”

**Published:** 1892-01

**Authors:** J. F. Eaves

**Affiliations:** Millican, Texas


For Daniel's Texas Medical Journal.

### LUCILIA MACELLARIA—"TEXAS SCREW WORM."

BY J. F. EAVES, M. D., MILLICAN, TEXAS.

THE Texas screw worm, as they are generally called, is one of the hardest of maggots to destroy. They have no pores and hence absorb nothing. They breathe by means of capillaries in the tail, and have to be destroyed by a very penetrating germicide. They generally attack the lower animals, but are occasionally met with in the tissues of man. Two years ago the writer spent about eight months experimenting with this worm in the lower animals.

There are three distinct varieties of *Lucilia Macellaria*. One deposited by the fly, hatches in a few minutes, if deposited in fresh blood; is of a yellowish brown color, tracheaform in appearance, with dark, raised bands running around his body; the bands are about 2 mm. apart, and are covered with black cillia.

Those of the second variety, after being deposited, require about 24 hours to hatch, and are just like the first variety, except the circular bands are not covered with cillia. They burrow continuously with the proboscis downwards, and are very quick in their motion.

The third variety are not tracheaform, are perfectly smooth, have a pinkish colored head, with a glistening white body. The proboscis resembles two small, black cillia, and is about one mm. in length. The eggs are never found, only on old wounds.

Several cases where this had hatched in man have come under my observation. A section-hand who had been drunk, came to me complaining of a continual tickling in his nose. The spores of the worm had been deposited in his nostrils while he was asleep; bloody mucous was continuously dropping from his nose, accompanied by the characteristic odor of a screw-worm sore.

I inserted a rubber tube into his mouth, carrying it as far into the pharynx as I could, without choking him ; then, dropping five or six drops of chloroform into the nostrils, directed the patient to inhale through the tube, holding the nostrils tight with my finger to prevent any air entering the nose, and exhale freely through the nose. In about ten minutes there dropped from his nostrils twenty-three well-grown screw worms. Patient was then directed to use a strong solution carbolic acid. He recovered without any trouble.

Chloroform is the only remedy I have found to act satisfactorily. Mercury seems to kill the worms, but is too slow for practical use in man. Calomel is, in my opinion, a dangerous remedy in the nasal cavity, and it is impossible to apply it to the post nasal cavity. Oil vitriol will kill them, but is too corrosive to be used in sufficient strength. I have tried everything of a germicidal nature, in the American pharmacopœia, and have found nothing so efficient as commercial chloroform. Mercurial ointment furnishes a good after-treatment, if the wound is on the skin.

